# Transcranial focused ultrasound precise neuromodulation: a review of focal size regulation, treatment efficiency and mechanisms

**DOI:** 10.3389/fnins.2024.1463038

**Published:** 2024-09-05

**Authors:** Jie Jin, Guangying Pei, Zhenxiang Ji, Xinze Liu, Tianyi Yan, Wei Li, Dingjie Suo

**Affiliations:** ^1^School of Medical Technology, Beijing Institute of Technology, Beijing, China; ^2^Advanced Research Institute of Multidisciplinary Science, Beijing Institute of Technology, Beijing, China

**Keywords:** neuromodulation, transcranial focused ultrasound, metasurfaces, ion channel, microbubbles

## Abstract

Ultrasound is a mechanical wave that can non-invasively penetrate the skull to deep brain regions to activate neurons. Transcranial focused ultrasound neuromodulation is a promising approach, with the advantages of noninvasiveness, high-resolution, and deep penetration, which developed rapidly over the past years. However, conventional transcranial ultrasound’s spatial resolution is low-precision which hinders its use in precision neuromodulation. Here we focus on methods that could increase the spatial resolution, gain modulation efficiency at the focal spot, and potential mechanisms of ultrasound neuromodulation. In this paper, we summarize strategies to enhance the precision of ultrasound stimulation, which could potentially improve the ultrasound neuromodulation technic.

## 1 Introduction

Neurological disorders pose a heavy burden on human health worldwide, and the corresponded interventions are urgently in need (Dayan et al., 2013). Neuromodulation is a promising approach in treating neurological disorders which developed rapidly over the past years. Currently, noninvasive neuromodulation techniques such as transcranial magnetic stimulation (TMS) (Hallett, 2007; Grossman et al., 2017), transcranial alternating current stimulation (tACS) (Grover et al., 2023) and transcranial direct current stimulation (tDCS) (Salling and Martinez, 2016), have been widely used in the treatment of neuropsychiatric disorders. However, spatial resolution and stimulation depth limit the applications of these techniques (Wagner et al., 2007; Romero et al., 2019). Transcranial focused ultrasound (tFUS) transmit a form of mechanical energy that could target the deep brain region non-invasively through the skull with a millimeter-sized focal spot (King et al., 2013). It has been widely used in medical applications for its advantages of high resolution, controllability, and noninvasiveness (Yoo et al., 2011; Pasquinelli et al., 2019; Landhuis, 2017; Bystritsky et al., 2011).

tFUS could modulate neurons without damage with energy level of I_SPTA_ < 500 mW/cm^2^ (Tyler et al., 2018). It has been applied in the modulation of brain activities in rodent (Li et al., 2016; Cui et al., 2019; Bobola et al., 2020; Tufail et al., 2010), rabbit (Yoo et al., 2011), pig (Dallapiazza et al., 2018), goat (Lee et al., 2016), monkey (Wattiez et al., 2017), and human (Legon et al., 2014). tFUS is also effective in treating neurological disorders such as consciousness, epilepsy, and obsessive-compulsive (Landhuis, 2017; Servick, 2020; Germann et al., 2021).

The spatial resolution of tFUS is crucial for achieving precise neuromodulation. To increase the tFUS resolution, acoustic metasurfaces offer a unique capability to customize wave fields, enabling complete control over phase and amplitude that could achieve subwavelength focus. Acoustic metasurfaces are 2D materials composed of subwavelength unit cells on millimeter and sub-millimeter scales, which could manipulate the wave fields freely. The modulation efficiency at the targeted region affects tFUS precision. Maximizing the modulation efficiency within the focal spot while minimizing the effect out of the focal spot is crucial to achieve precise modulation. tFUS with appropriate stimulation parameters could increase the modulation efficiency of the targeted region. The mechanism of tFUS neuromodulation remains unclear (Yoo et al., 2022). The mechanosensitive ion channels could transform mechanical stimuli into electric or chemic signals under tFUS (Jin et al., 2020). There are a few mechanosensitive ion channels could be activated by tFUS which hold the potential for tFUS neuromodulation.

In this review, we focus on techniques aimed at enhancing the precise of neuromodulation. Firstly, we explore the potential of metasurfaces to increase resolution by minimizing the focal spot size of tFUS. Secondly, different approaches of gaining modulation efficiency of the tFUS focal spot are investigated, such as the optimization of tFUS parameters and the application of microbubbles. Thirdly, mechanisms underlying tFUS neuromodulation are reviewed, with a particular emphasis on the involvement of mechanosensitive ion channels in refining targeted modulation.

## 2 Focal size regulation of tFUS

The resolution of tFUS affects the accuracy of neuromodulation. Smaller focal spot could minimize the impact outside the targeted region. Therefore, tFUS with high resolution and enough focal intensity is urgently needed.

**Figure 1:**
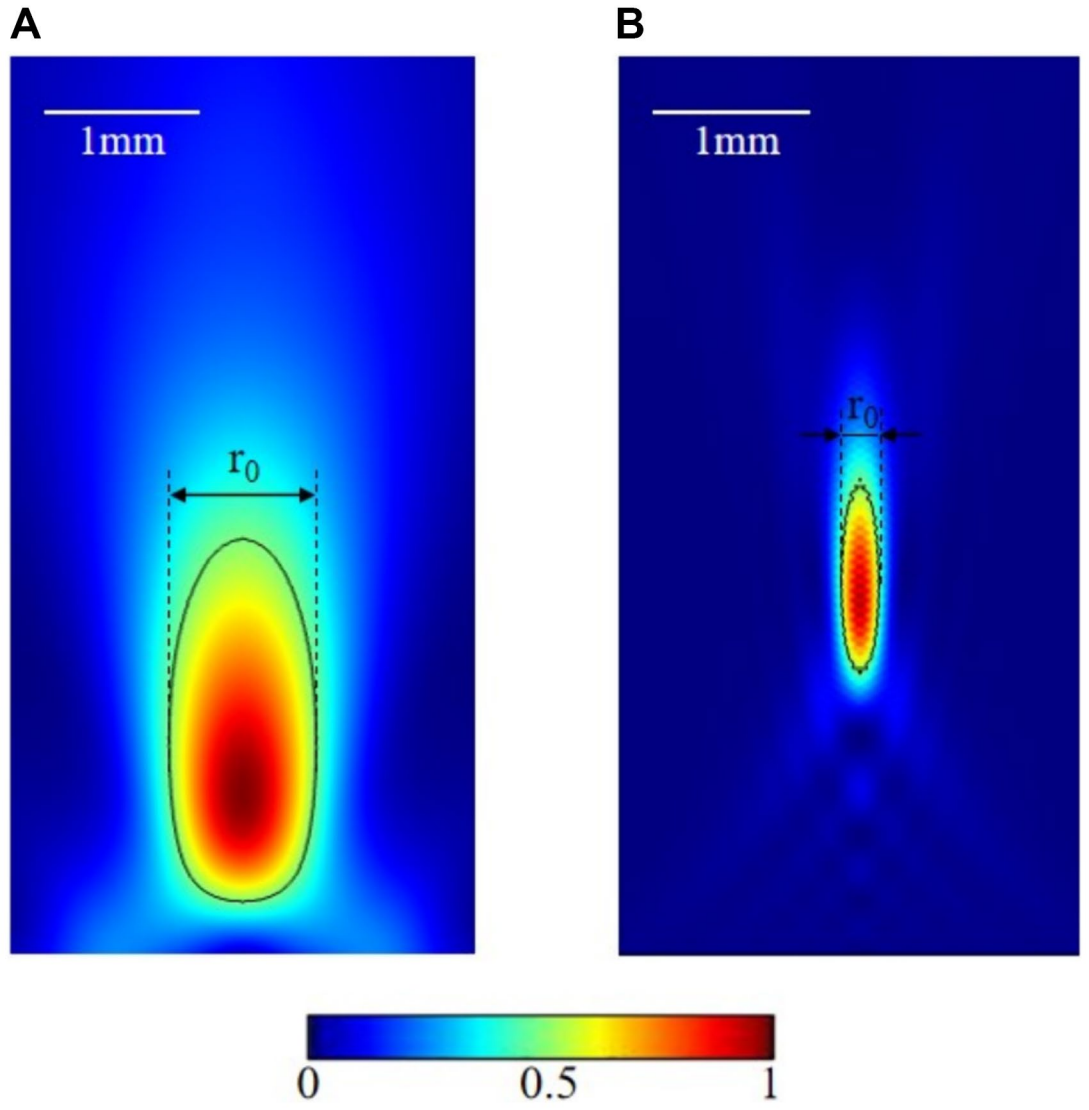
Focusing effect at different frequencies. **(A)** 1 MHz transducer focus. **(B)** 5 MHz transducer focus.

The most popular focusing methods are through geometrical curve (Marzo et al., 2018), lens (Sanchis et al., 2010), and phased array (Marzo et al., 2015). The size of the focal spot lies in millimeter range depends on the size and frequency of the focused transducer. Increasing the fundamental frequency or the transducer aperture could increase the resolution. As shown in Figure 1A, the axial focusing of the transducer with aperture 3.4 cm and focal length 1.7 cm is 1 mm at 1 MHz frequency. While increasing the frequency to 5 MHz, axial focusing could be reduced to 0.25 mm (Figure 1B). However, the high frequency could introduce extra thermal effect, attenuation, and scattering in transcranial applications, thus, reducing the efficiency of this promising technic. Standard frequencies of tFUS are less than 1 MHz.

Acoustic metasurfaces is able to achieve subwavelength focal spots in different applications. Three types of metasurfaces—refractive, diffractive, and reflective—have been proposed to realize acoustic focusing. Refractive metasurfaces could attain specific focusing functions based on the effective distribution of the acoustic refractive index. Zhou et al. introduced a solid phononic crystal lens that achieved super-focusing capabilities beyond the diffraction limit (Zhou et al., 2014). Jin et al. reported a class of acoustic gradient-index metasurfaces engineered from soft graded-porous silicone rubber, which could realize beam steering and beam focusing (Jin et al., 2019). Reflective metasurfaces converge reflected waves by calculating gradient phases induced by surface unites at the designed focal spot. Qi et al. proposed multilateral metasurfaces that had excellent performance in acoustic energy confinement (Qi et al., 2017). Wu et al. proposed a broadband metasurface that reflects US waves to enhance the focus effect (Wu et al., 2016). Diffractive metasurfaces, including grating lobes and Fresnel zone plate (FZP) types, are utilized to manipulate the patterns of US waves. Chiang et al. developed a metalens capable of narrowing the focal spot to 0.364 times the wavelength (Chiang et al., 2021). Astolfi et al. demonstrated the construction of air-filled polymer shell lenses utilizing evenly-spaced concentric rings. The lens achieved a full width at half maximum (FWHM) of 0.65 wavelengths at the focal spot (Astolfi et al., 2022).

**Figure 2:**
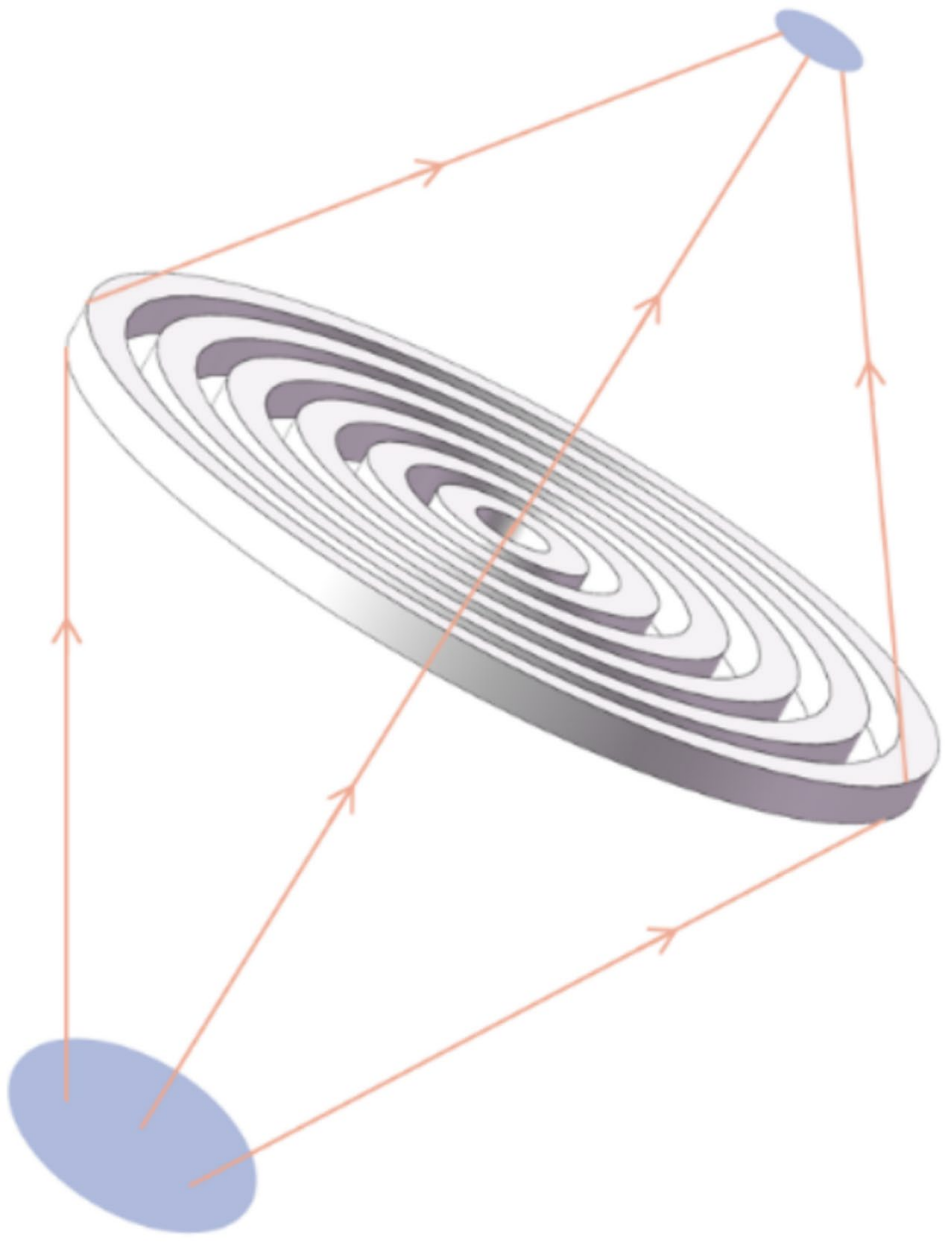
Schematic diagram of the FZP focusing lens.

The FZP lens exhibits excellent focusing performances such as the focal spot gain for its concentric circle structure comprising adjacent transparent zones (Figure 2; Tarrazó-Serrano et al., 2019; Xia et al., 2020; Calvo et al., 2015). The FZP lens has the capability to adjust the number of rings, thickness, and width to modulate the size of the focal spot. Chen et al. created an acoustic hypersurface lens composed of a group of deep sub-wavelength-scale slit structures that could modulate far-field and near-field acoustic focusing simultaneously (Chen et al., 2018). Meanwhile, Ma et al. utilized a time-reversal technique to accomplish acoustic focusing. After that, an open-cavity structure with sub-wavelength dimensions was introduced at the focal spot to confine the acoustic wave energy inside (Ma et al., 2020). Pan et al. proposed the Soret type of the FZP lens, which demonstrates a subwavelength underwater sound focusing effect across a broad frequency band (Pan et al., 2023). Jiang et al. addressed limitations by enlarging the feature size of the metamaterial while maintaining a compact overall geometry, achieving a robust subwavelength US focus. This enhancement is achieved by increasing the acoustic field energy and reducing the focal spot size effectively, enabling sub-wavelength focusing. The realization of a sub-wavelength focal spot can be facilitated using a lens to create a “coarse to fine” effect (Jiang et al., 2022; Tarrazó-Serrano et al., 2019). Shin et al. demonstrated a sub-wavelength acoustic focus using a planar US transducer equipped with a metasurface piezoelectric annular array, achieving a needle-like sub-wavelength focus (Hur et al., 2022).

Acoustic metasurfaces provide a versatile platform for controlling the acoustic field with high precision. Hu et al. designed the Airy-beam holographic, which enables precise and free ultrasound neuromodulation (Hu et al., 2024). The acoustic metasurfaces allow for the reduction of transducer size, focal length, and multi-point focusing capabilities. Metasurfaces offer unique acoustic properties compared with conventional methods, enabling the development of a wide range of novel acoustic devices with diverse functions. They aim at new opportunities for achieving high-precision, high-performance, and cost-effective integrated acoustic focusing. Thus, optimizing the focusing performance through metasurfaces is essential for achieving targeted neuromodulation with tFUS.

## 3 Enhancement of modulation efficiency

Neurons located outside the targeted region may be stimulated by US, which impairs stimulation accuracy. Therefore, achieving the focal spot modulation efficiency and minimizing the effect of tFUS outside the focal spot is crucial for enhancing the spatial resolution of tFUS neuromodulation.

### 3.1 Increasing modulation efficiency based on microbubbles

**Figure 3:**
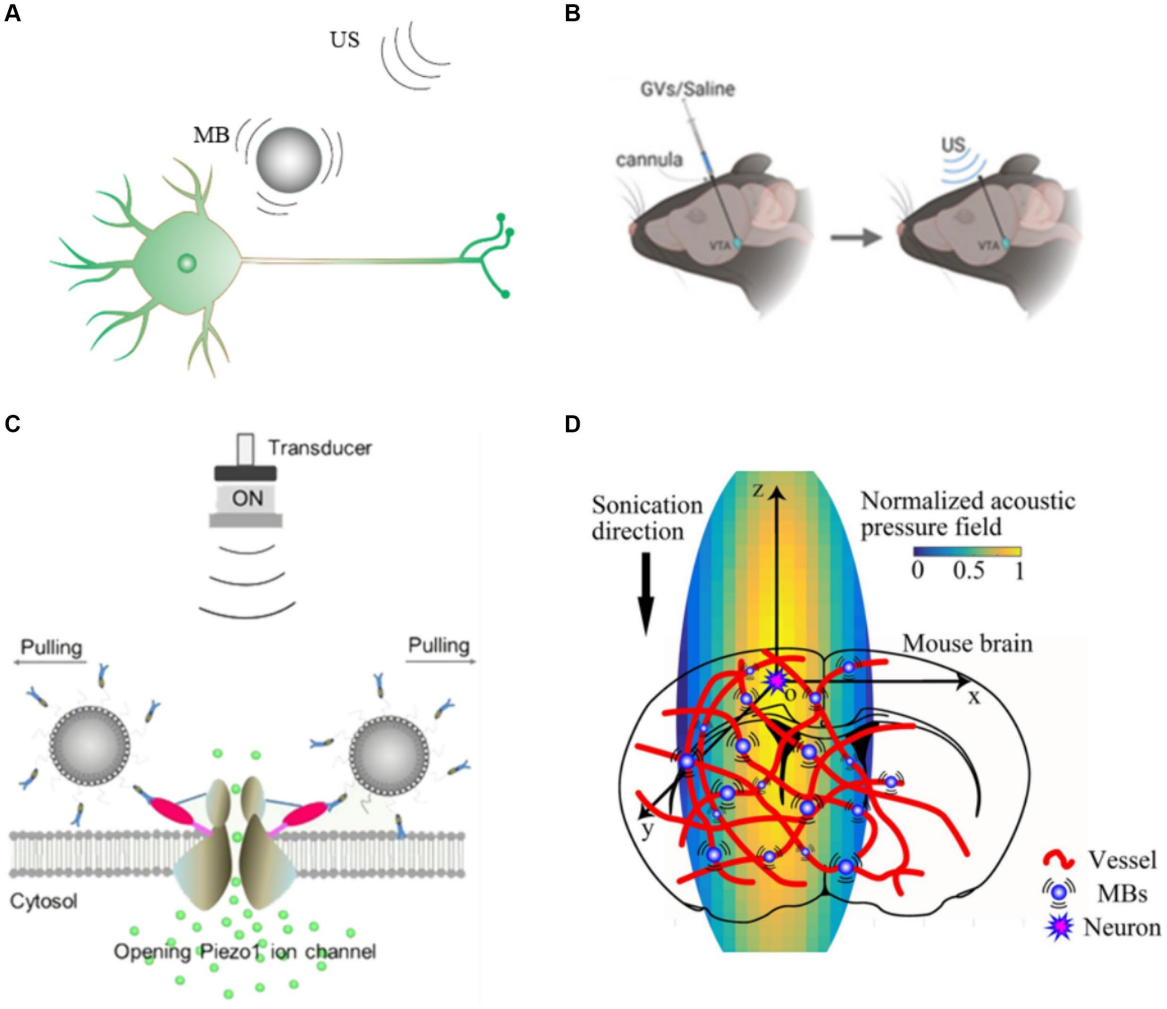
Gain modulation efficiency based microbubbles **(A)** US combined with microbubble modulation. **(B)** Schematic illustration of our GVs/saline injection and US stimulation plan (Hou et al., 2021). **(C)** Diagram of PTMB binding to the cells and the enhanced calcium ion influx by US stimulation (Shen et al., 2021). **(D)** Schematic of the mice cortex model (Cui et al., 2020).

Microbubbles are sensitive to US waves and are widely used in US contrast imaging. Researches indicate that oscillating microbubbles could exert force on the surrounding medium (Yildiz et al., 2022). Microbubbles undergo stable cavitation at low acoustic pressure. The oscillating microbubbles generate scattered force (Figure 3A) that enhances the acoustic radiation force and increases the stimulation success rate of US without harming tissues (Meng et al., 2019).

Microbubble mediated neuromodulation has shown the potential to amplify the treatment efficiency. Ibsen et al. modulated nematode neurons under the body surface cuticle using US combined with microbubbles. US without microbubbles did not activate the nematode neuron, and US combined with microbubbles activated this neuron because the mechanical deformations produced by the oscillating microbubbles were transmitted to the nematode neuron (Ibsen et al., 2015). Cui et al. injected microbubbles into the tail vein of mice and stimulated the motor cortex (M1) with tFUS, which significantly enhanced the c-Fos expression. The combination of US and microbubbles under low acoustic pressure increased the success rate of stimulation without causing tissue damage (Cui et al., 2020). Harriet et al. demonstrated that nanodroplets by tail vein injection could be used for both neuroinhibition and neurostimulation without disrupting the blood–brain barrier of the rat using US stimulation (Lea-Banks et al., 2021).

Surface modification of microbubbles or other means of injecting microbubbles into targeted region could significantly improve the stimulation accuracy. Hou et al. utilized US driven gas vesicles (GVs) to achieve precise, reversible and reproducible neuromodulation (Figure 3B; Hou et al., 2021). GVs is nano-sized protein structures extracted from cyanobacteria, which have similar acoustic properties to microbubbles. The resolution of US stimulation could be increased because nearby neurons with GVs could be activated by lower acoustic pressure US stimulation. Shen et al. (Figure 3C) developed piezo1-targeted microbubbles (PTMB) that could bind to piezo1 channels (Figure 3D), and US causes the microbubbles to oscillate so that the neurons could be activated at lower US acoustic whereas neurons not bound to the PTMB require higher acoustic pressure to be activated (Shen et al., 2021). This approach achieves targeted US stimulation and provides an effective strategy for precise neuromodulation.

US-excited microbubbles are a strategy for effectively targeting and amplifying US stimulation. However, microbubbles need to be injected through the vein, and then the microbubbles circulate in the bloodstream with low targetability. Injecting microbubbles directly into the corresponding brain regions requires craniotomy and is not considered a safe or clinically translatable option. It is feasible to change the surface of microbubble shells and apply modifications to the molecules or antibodies using genetic engineering.

### 3.2 Increasing modulation efficiency based on ultrasound parameters

**Figure 4:**
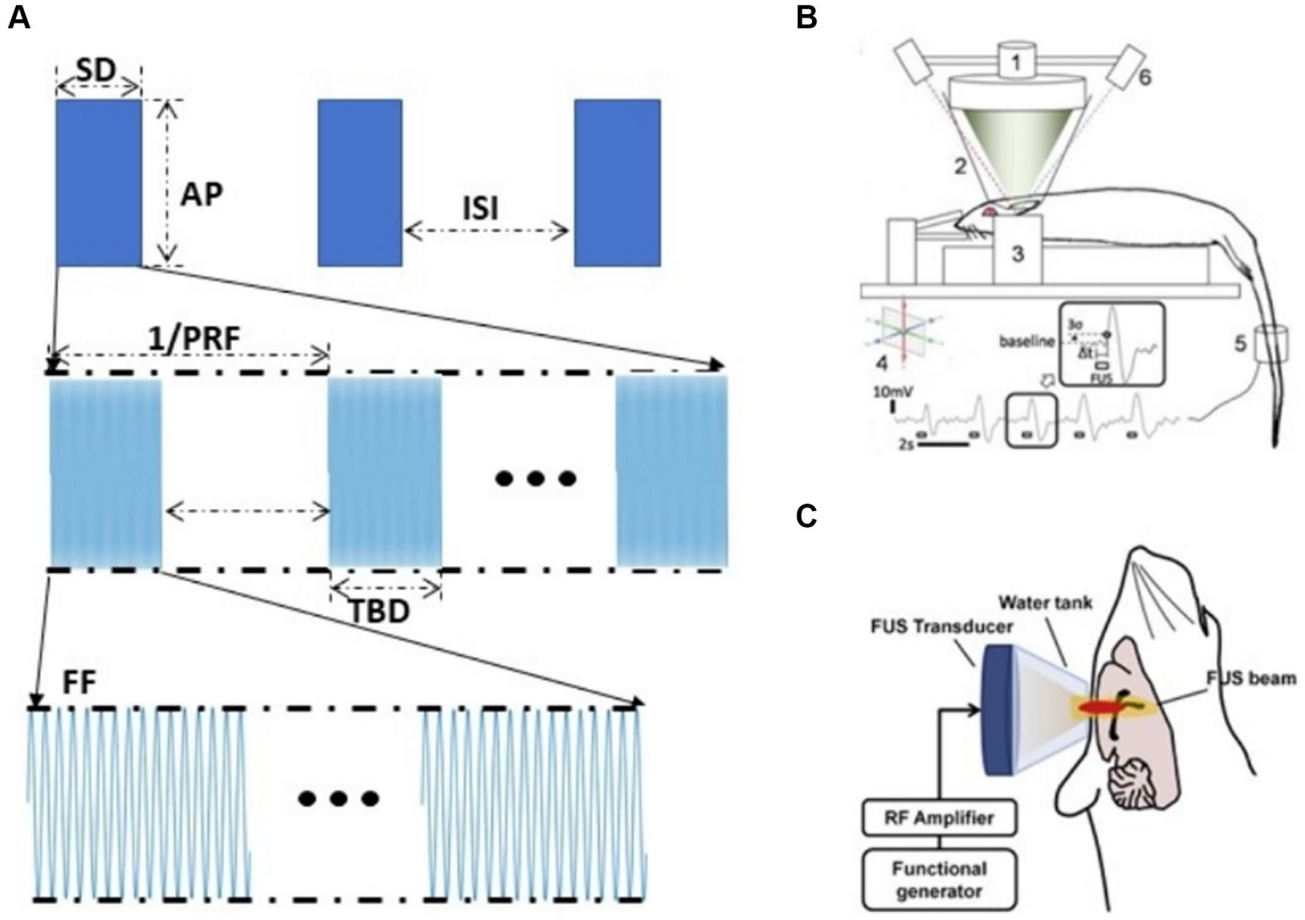
Gain modulation efficiency based on US parameters **(A)** US parameters. **(B)** Experimental set-up to test excitatory neuromodulation using FUS in rat (Kim et al., 2014). **(C)** tFUS setup for epilepsy therapy (Chen et al., 2020).

The effect of tFUS neuromodulation depends on stimulation parameters (Wang et al., 2020). Pulsed US parameters include five parameters: fundamental frequency (FF), pulse repetition frequency (PRF), duty cycle (DC), stimulation duration (SD), and acoustic pressure (AP) (O'brien, 2007; Figure 4A). Different tFUS studies have shown that excitatory or inhibitory of US might vary with the experimental conditions (Yu et al., 2016). Therefore, appropriate tFUS parameters could significantly enhance the effect of neuromodulation (Tufail et al., 2010; Mueller et al., 2014; Lee et al., 2015; King et al., 2013). Fomenko et al. investigated the effect of different acoustic parameters on the amplitude of motor evoked potentials (MEP), it demonstrated that DC had a significant effect on MEP amplitude, with 10% DC inhibiting it, while 30% DC had no effect. In addition, stimulation duration also had a similarly significant effect on MEP amplitude according to their research (Fomenko et al., 2020). Park et al. investigated the effect of stimulating the medial prefrontal cortex (mPFC) using unfocused US at 40 Hz PRF and 300 kHz FF could reduce Aβ plaques and enhance brain connectivity (Park et al., 2021). Kim et al. utilized US with 70% DC and 5 s interstimulus interval (ISI) to activate bilateral mPFC, whereas utilizing 5% DC with no interstimulus interval inhibited the mPFC (Kim et al., 2022). Kim et al. found that at 300 s SD, 350 kHz FF, 50% DC stimulation of rat M1 was superior to motor responses elicited by 30 and 70% DC stimulation; at 50% DC, 1–5 ms TBDs, 350 kHz FF was superior to 650 kHz, and pulsed tFUS was superior to equivalent continuous tFUS (Figure 4B; Kim et al., 2014). King et al. used US to elicit a motor response in mice and found that the success rate of elicitation increased with increasing AP and SD (King et al., 2013). Ye et al. investigated that with increasing FF, greater AP was required to achieve the same motor response (Ye et al., 2016). Yu et al. demonstrated that tFUS was able to induce neuronal activation *in vivo* and that the degree of neuronal response and the time to activation was strongly correlated with AP and SD, with SD of 0.4 s, 0.5 s were inhibition effects, while 0.1, 0.2, and 0.3 s were no effects (Yu et al., 2016).

Adjusting the parameters of tFUS stimulation could produce excitatory or inhibitory effects on the central nervous system at the stimulated region, which could be reversibly neuromodulated. Chen et al.’s study (Figure 4C) showed that US could inhibit pentylenetetrazol which induces abnormal neuron discharges in acutely epileptic rats and the effect of tFUS on elilepsy suppression is related to the choice of parameters. Sharabi et al. used a 230 kHz spherical phased array US that effectively suppressed tremors in Parkinsonian rats and was able to induce motor responses in the tail and leg (Sharabi et al., 2019). According to Yoon et al. the tFUS stimulation with higher DC (>30%) and shorter SD favored activation of the targeted brain region, whereas stimulation with lower DC (<10%) and longer SD (>1 min) inhibited the activity of the targeted brain regions (Yoon et al., 2019).

Enhancing modulation efficiency is another effective strategy to increase the spatial resolution of tFUS neuromodulation. The scattered pressure of the oscillating microbubbles on the surrounding medium and the optimization of the parameters to increase the sensitivity of the tissue to tFUS could enhance the modulation efficiency. Therefore, microbubble and tFUS parameters could be considered in improving tFUS neuromodulation accuracy.

## 4 Mechanisms of tFUS neuromodulation

**Figure 5:**
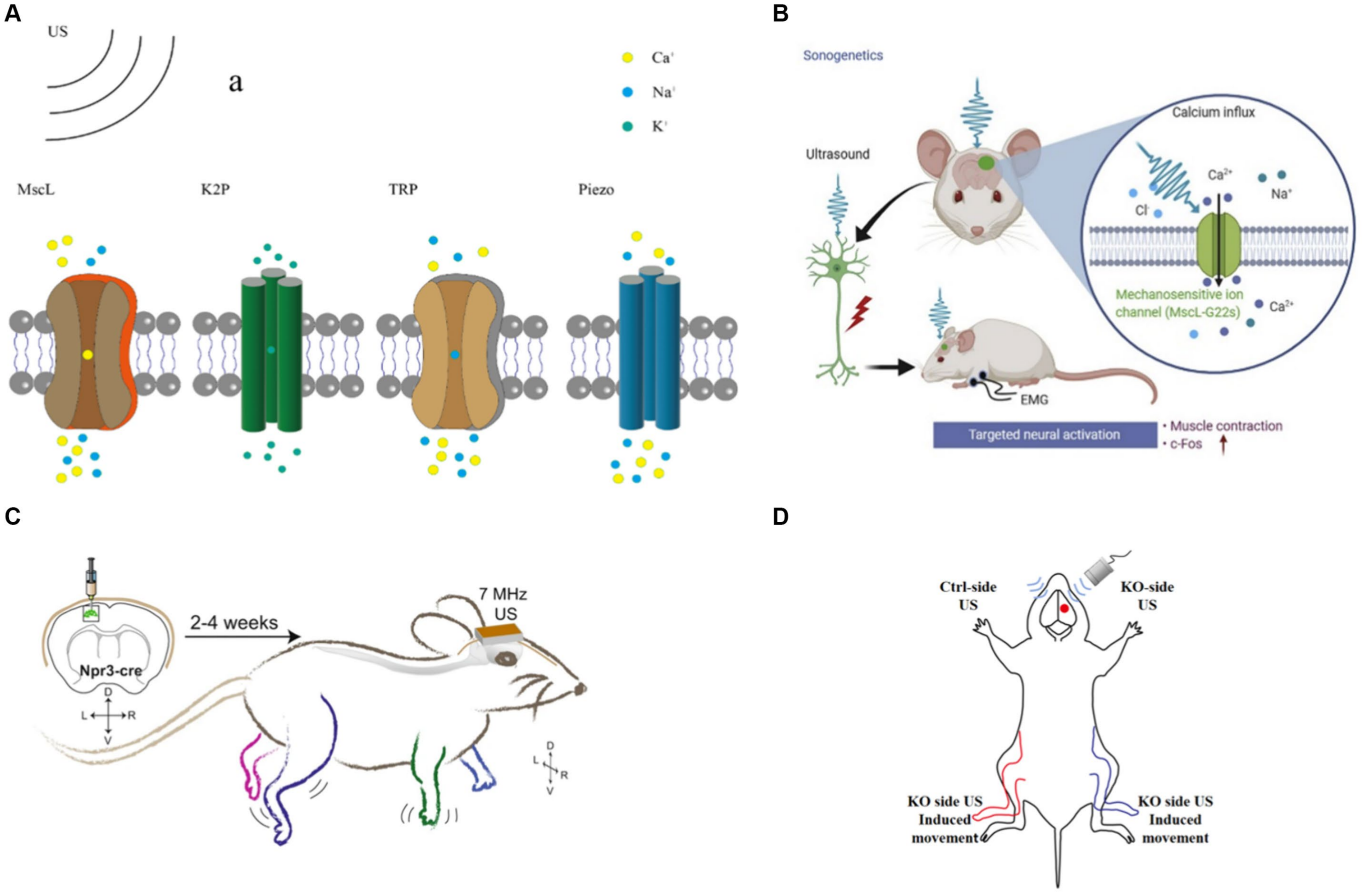
tFUS neuromodulation **(A)** tFUS neuromodulation based on mechanosensitive channel. **(B)** US targeted at the cortical M1 region with MscL evoked rapid EMG responses (Qiu et al., 2021). **(C)** US modulation of mice M1 expressing hsTRPA1 (Duque et al., 2022). **(D)** US stimulate Ctrl-side and KO-side.

Ion channel, protein expressed by living cells that provides a pathway for charged ions from dissolved salts, including sodium, calcium, potassium, and chloride ions, to pass through the otherwise impermeable lipid cell membrane (Ye et al., 2018). One of the mechanisms of tFUS neuromodulation is based on mechanosensitive ion channels (Tyler, 2011). tFUS could activate mechanosensitive ion channels in neurons (Figure 5A), alter the permeability of cell membranes, and modulate intracellular ion concentrations (Meng et al., 2019). To achieve targeted tFUS neuromodulation, genetic manipulation techniques are employed to express mechanical force-sensitive receptors on neurons. This confers cellular sensitivity to US stimulation, which acquires specificity for the cell or tissue. This method is the most studied means of targeted tFUS neuromodulation.

### 4.1 MscL

Mechanosensitive Channel of Large Conductance (MscL) is widely expressed in prokaryotic cells (Martinac et al., 1987). The state of MscL is regulated by membrane tension. MscL allow the passage of small molecules such as potassium ions, sodium ions, and glucose.

Research has demonstrated the ability of US stimulation to activate MscL. Following the successful expression of MscL in mammalian cells by Doerner (Doerner et al., 2012), Ye et al. expressed MscL in primary cultured rat hippocampal neurons and utilized US at 29.92 MHz frequency to activate MscL and achieve neuronal modulation. MscL expression did not alter the original electrophysiological properties or survival of the neurons (Ye et al., 2018).

Qiu et al. expressed MscL-G22S *in vitro* and induced calcium influx and neuronal activation using 500 kHz US. Subsequently, they successfully expressed MscL-G22S in the primary M1 of mice under the same FF of US stimulation as in the *in vitro* experiments. They observed that MscL-expressed mice exhibited an increased magnitude of electromyography (EMG) response and success rate. Additionally, M1 neurons showed higher expression levels of c-Fos (Figure 5B; Qiu et al., 2021). The MscL responded to a wide range of US frequencies, ranging from 0.5 MHz to 30 MHz, and they could be activated by lower intensity US, which is conducive to achieving tFUS neuromodulation.

### 4.2 TRP

Transient receptor potential (TRP) channels are widely distributed ion channel proteins found in the cell membranes of various organisms, playing crucial roles in numerous cellular physiological and pathological processes. Ibsen et al. discovered that US combined with microbubbles could modulate the locomotor behavior of *Caenorhabditis elegans* expressed TRP-4 channels. They further observed an increase in calcium accumulation in TRP-4-expressing AWC neurons stimulated by US combined with microbubbles, indicating the activation of TRP channels by US (Ibsen et al., 2015). However, TRP-4 channels are not effective in mammals. To enhance the targeting of US, the team stimulated HEK cells *in vitro* using US at a frequency of 7 MHz, resulting in increased calcium levels and activation of HEK cells (Figure 5C; Duque et al., 2022). Additionally, *in vitro* stimulation of the M1 of mice expressing TRPA1 channels led to significant electromyographic responses in the corresponding limbs and increased neuronal c-Fos expression. Notably, no significant increase in c-Fos expression was detected in the auditory cortex, suggesting that US neuromodulation does not involve the auditory pathway (Sato et al., 2018; Guo et al., 2018). Yang et al. induced hibernation-like hypothermia and hypometabolic state in rats by applying US stimulation to the preoptic area (POA) (Yang et al., 2023). Knockdown of TRPM2 channels in POA neurons inhibited US-induced hypothermia and hypometabolic behavior. Furthermore, Yang et al. expressed TRPV1 channels in the primary somatosensory cortex of mice and utilized US to activate the TRPV1 channel. They concluded that TRPV1 channel activation was attributed to the thermal response induced by US (Yang et al., 2023). As TRPV1 channels are activated at temperatures of 42°C or higher, the thermal effect of US could be employed to gate TRPV1 channels (Kwon et al., 2022). TRP channels exhibit responsiveness to US stimulation across a wide range of frequencies and could be gated by US stimulation. However, these channels typically require high acoustic pressure for activation. Mutation scanning protein engineering techniques could be utilized in future studies to increase the sensitivity of TRP channels to lower acoustic pressure US stimulation.

### 4.3 K2P

Two-pore domain potassium (K2P) channels, also known as biportal ion channels, are specific types of ion channels used for potassium ion transport in cell membranes (Fink et al., 1998). The TRAAK channels are categorized within K2P channels family, with their activity regulated by membrane tension (Brohawn et al., 2014). Kubanek et al. expressed TREK-1, TREK-2, and TRAAK channels of the K2P family in African Xenopus oocytes. Using US to stimulate the cell while detecting electrophysiological signals, it was found that US stimulation could regulate the activity of K2P channels and alter the membrane potential (Kubanek et al., 2016). Sorum et al. expressed TRAK channels in African Xenopus oocytes and found increased cell membrane tension and activated TRAK channels by increasing AP. It is shown that US could activate TRAK channels and the activation state is related to AP (Sorum et al., 2021). In general, US could activate K2P channels, but requires a higher US FF and AP.

### 4.4 Pizeo

Piezo channels are widely expressed in mammals and have extensive distribution in humans (Coste et al., 2010). To date, two types of ion channels, Piezo1 and Piezo2, have been identified. Piezo1 is the most extensively studied channel in the context of US modulation. Piezo1 stands out as one of the most sensitive mechanosensitive ion channels, capable of activation by forces exceeding 10 pN (Wu et al., 2016).

Qiu et al. employed US to activate Piezo1 channels expressed in HEK-293 T cells and in primary neurons of mice. Their findings demonstrate that US alone is capable of activating both exogenous and endogenous Piezo1 channels (Qiu et al., 2019). Pan et al. expressed Piezo1 channels in HEK293T cells and utilized microvesicle-mediated inward calcium ion flow to trigger downstream pathways by inducing the opening of Piezo1 ion channels with US (Pan et al., 2018). Zhu et al. demonstrated that Piezo1 knockout (P1KO) in the M1 of mice resulted in a significant decrease in US-induced limb movement and electromyography (EMG) responses, highlighting the critical role of Piezo1 channels as key mediators of tFUS neuromodulation (Figure 5D; Zhu et al., 2023). Liao et al. reported that Piezo1 is activated in response to US-induced shear stress, leading to an elevation in intracellular calcium ion levels (Liao et al., 2021). Li et al. identified the essential role of Piezo2-mediated hearing in mice, suggesting that Piezo2 channels could be gated by US stimulation (Li et al., 2021).

Ion channel-based modulation currently represents the most effective means of achieving targeted, high-precision stimulation using US. Furthermore, it remains the most extensively studied method in this field. Nevertheless, certain mechanosensitive ion channels, such as TREK-1, TREK-2, and TRAAK, necessitate a high frequency US and present challenges in clinical application. US-targeted neuromodulation with the help of ion channels requires that the target ion channels be expressed on neurons by AAV virus, which is mainly realized by stereotactic brain injection, requiring craniotomy for virus injection and low safety. With the development of genetic engineering, studies have been conducted to modify the AAV virus capsid so that it could effectively cross the blood–brain barrier and target specific cells in the brain (Goertsen et al., 2022). This enables the expression of specific genes in particular neurons through intravenous injection of the virus (Table 1).

**Table 1:** Parameters of ultrasound-activated mechanosensitive ion channels.

Channel	**Frequency** (MHz)	**Sound pressure** (MPa)	**Duty cycle** (DC)	Pulse repetition frequency (PRF)	Citation
MscL					
MscL-G22S	10	0.095	25%	5 Hz	Heureaux et al. (**2014**)
MscL-I92L	29.92	0.45	/	/	Ye et al. (**2018**)
MscL-G22S	0.5	0.05–0.35	40%	1 kHz	Qiu et al. (**2021**)
TRP					
TRP-4	2.25	0.9	Continuous waves	Continuous waves	Ibsen et al. (2015)
TRPA1	7	2.5	Continuous waves	Continuous waves	Duque et al. (2022)
TRPA1	0.43	0.673	50%	2 kHz	Oh et al. (2019)
TRPP1	0.3	/	Continuous waves	Continuous waves	Yoo et al. (2022)
TRPP2	0.3	/	Continuous waves	Continuous waves	Yoo et al. (2022)
TRPM2	3.2	1.6	20%	10 Hz	Yang et al. (2023)
K2P					
TREK-1	10	/	Continuous waves	Continuous waves	Kubanek et al. (2016)
TREK-2	10	/	Continuous waves	Continuous waves	Kubanek et al. (2016)
TRAAK	10	/	Continuous waves	Continuous waves	Kubanek et al. (2016)
TRAAK	4.78	/	Continuous waves	Continuous waves	Sorum et al. (2021)
Piezo					
Piezo1	0.5	0.3	40%	1 kHz	Qiu et al. (2019)
Pizeo1	2	0.17	50%	1 kHz	Shen et al. (2021)
Pizeo1	2	0.6	10%	5 Hz	Zhang et al. (2020)
Pizeo1	0.3, 0.35, 0.4, 0.45	0.3–0.45	50%	1 kHz	Zhu et al. (2023)

## 5 Conclusion

In summary, tFUS neuromodulation combines noninvasiveness, high spatial resolution, and depth penetration. It has experienced a rapid expansion over the past years and holds significant promise for treating neurological disorders and elucidating neural circuits. Current efforts are being directed at exploring which ways to maximize the precision of tFUS stimulation since it impacts the effectiveness of modulation.

In this paper, we investigated the impact of focal size, treatment efficiency and mechanism on enhancing tFUS modulation accuracy. All reviewed methods have improved resolution and efficiency, with each type of method improving differently. However, in general, there are still shortcomings in terms of focus accuracy and security. Combining acoustic metasurfaces and ion channel and selecting appropriate stimulation parameters is an effective means of achieving precise ultrasound neuromodulation.

## References

[ref1] Astolfi L. Hutchins D. A. Watson R. L. Thomas P. J. Ricci M. Nie L.. (2022). Optimised polymer trapped-air lenses for ultrasound focusing in water exploiting Fabry-Pérot resonance. Ultrasonics 125:106781. doi: 10.1016/j.ultras.2022.106781, PMID: 35671568

[ref2] Bobola M. S. Chen L. Ezeokeke C. K. Olmstead T. A. Nguyen C. Sahota A.. (2020). Transcranial focused ultrasound, pulsed at 40 Hz, activates microglia acutely and reduces Aβ load chronically, as demonstrated in vivo [J]. Brain Stimul. 13, 1014–1023. doi: 10.1016/j.brs.2020.03.016, PMID: 32388044 PMC7308193

[ref3] Brohawn S. G. Campbell E. B. Mackinnon R. (2014). Physical mechanism for gating and mechanosensitivity of the human TRAAK K+ channel. Nature 516, 126–130. doi: 10.1038/nature14013, PMID: 25471887 PMC4682367

[ref4] Bystritsky A. Korb A. S. Douglas P. K. Cohen M. S. Melega W. P. Mulgaonkar A. P.. (2011). A review of low-intensity focused ultrasound pulsation. Brain Stimul. 4, 125–136. doi: 10.1016/j.brs.2011.03.007, PMID: 21777872

[ref5] Calvo D. C. Thangawng A. L. Nicholas M. Layman C. N. (2015). Thin Fresnel zone plate lenses for focusing underwater sound. Appl. Phys. Lett. 107:014103. doi: 10.1063/1.4926607

[ref6] Chen S. G. Tsai C. H. Lin C. J. Lee C. C. Yu H. Y. Hsieh T. H.. (2020). Transcranial focused ultrasound pulsation suppresses pentylenetetrazol induced epilepsy in vivo. Brain Stimul. 13, 35–46. doi: 10.1016/j.brs.2019.09.011, PMID: 31575487

[ref7] Chen J. Xiao J. Lisevych D. Shakouri A. Fan Z. (2018). Deep-subwavelength control of acoustic waves in an ultra-compact metasurface lens. Nat. Commun. 9:4920. doi: 10.1038/s41467-018-07315-6, PMID: 30467347 PMC6250707

[ref8] Chiang Y. K. Quan L. Peng Y. Sepehrirahnama S. Oberst S. Alù A.. (2021). Scalable Metagrating for efficient ultrasonic focusing. Physical Rev. Appl. 16:064014. doi: 10.1103/PhysRevApplied.16.064014

[ref9] Coste B. Mathur J. Schmidt M. Earley T. J. Ranade S. Petrus M. J.. (2010). Piezo1 and Piezo2 are essential components of distinct mechanically activated cation channels. Science 330, 55–60. doi: 10.1126/science.1193270, PMID: 20813920 PMC3062430

[ref10] Cui Z. Li D. Feng Y. Xu T. Wu S. Li Y.. (2019). Enhanced neuronal activity in mouse motor cortex with microbubbles' oscillations by transcranial focused ultrasound stimulation. Ultrason. Sonochem. 59:104745. doi: 10.1016/j.ultsonch.2019.104745, PMID: 31473423

[ref11] Cui Z. Li D. Xu S. Xu T. Wu S. Bouakaz A.. (2020). Effect of scattered pressures from oscillating microbubbles on neuronal activity in mouse brain under transcranial focused ultrasound stimulation. Ultrason. Sonochem. 63:104935. doi: 10.1016/j.ultsonch.2019.104935, PMID: 31945558

[ref12] Dallapiazza R. F. Timbie K. F. Holmberg S. Gatesman J. Lopes M. B. Price R. J.. (2018). Noninvasive neuromodulation and thalamic mapping with low-intensity focused ultrasound. J. Neurosurg. 128, 875–884. doi: 10.3171/2016.11.JNS16976, PMID: 28430035 PMC7032074

[ref13] Dayan E. Censor N. Buch E. R. Sandrini M. Cohen L. G. (2013). Noninvasive brain stimulation: from physiology to network dynamics and back. Nat. Neurosci. 16, 838–844. doi: 10.1038/nn.3422, PMID: 23799477 PMC4876726

[ref14] Doerner J. F. Febvay S. Clapham D. E. (2012). Controlled delivery of bioactive molecules into live cells using the bacterial mechanosensitive channel MscL. Nat. Commun. 3:990. doi: 10.1038/ncomms1999, PMID: 22871809 PMC3651673

[ref15] Duque M. Lee-Kubli C. A. Tufail Y. Magaram U. Patel J. Chakraborty A.. (2022). Publisher correction: sonogenetic control of mammalian cells using exogenous transient receptor potential A1 channels. Nat. Commun. 13:1130. doi: 10.1038/s41467-022-28838-z, PMID: 35217673 PMC8881586

[ref16] Fink M. Lesage F. Duprat F. Heurteaux C. Reyes R. Fosset M.. (1998). A neuronal two P domain K+ channel stimulated by arachidonic acid and polyunsaturated fatty acids. EMBO J. 17, 3297–3308. doi: 10.1093/emboj/17.12.3297, PMID: 9628867 PMC1170668

[ref17] Fomenko A. Chen K. H. S. Nankoo J. F. Saravanamuttu J. Wang Y. el-Baba M.. (2020). Systematic examination of low-intensity ultrasound parameters on human motor cortex excitability an behavior. eLife 9:9. doi: 10.7554/eLife.54497 PMC7728443 33236981

[ref18] Germann J. Elias G. J. B. Neudorfer C. Boutet A. Chow C. T. Wong E. H. Y.. (2021). Potential optimization of focused ultrasound capsulotomy for obsessive compulsive disorder. Brain 144, 3529–3540. doi: 10.1093/brain/awab232, PMID: 34145884

[ref19] Goertsen D. Flytzanis N. C. Goeden N. Chuapoco M. R. Cummins A. Chen Y.. (2022). AAV capsid variants with brain-wide transgene expression and decreased liver targeting after intravenous delivery in mouse and marmoset. Nat. Neurosci. 25, 106–115. doi: 10.1038/s41593-021-00969-4 34887588

[ref20] Grossman N. Bono D. Dedic N. Kodandaramaiah S. B. Rudenko A. Suk H. J.. (2017). Noninvasive deep brain stimulation via temporally interfering electric fields. Cell 169, 1029–41.e16. doi: 10.1016/j.cell.2017.05.024, PMID: 28575667 PMC5520675

[ref21] Grover S. Fayzullina R. Bullard B. M. Levina V. Reinhart R. M. G. (2023). A meta-analysis suggests that tacs improves cognition in healthy, aging, and psychiatric populations. Sci. Transl. Med. 15:eabo2044. doi: 10.1126/scitranslmed.abo2044, PMID: 37224229 PMC10860714

[ref22] Guo H. Hamilton Ii M. Offutt S. J. Legon W. Alford J. K. Lim H. H. (2018). Ultrasound produces extensive brain activation via a Cochlear pathway. Neuron 99:866. doi: 10.1016/j.neuron.2018.07.049, PMID: 30138592

[ref23] Hallett M. (2007). Transcranial magnetic stimulation: a primer. Neuron 55, 187–199. doi: 10.1016/j.neuron.2007.06.026, PMID: 17640522

[ref24] Heureaux J. Chen D. Murray V. L. Deng C. X. Liu A. P. (2014). Activation of a bacterial mechanosensitive channel in mammalian cells by cytoskeletal stress. Cell. Mol. Bioeng. 7, 307–319. doi: 10.1007/s12195-014-0337-8, PMID: 25606062 PMC4297646

[ref25] Hou X. D. Qiu Z. H. Xian Q. X. Kala S. Jing J. Wong K. F.. (2021). Precise ultrasound Neuromodulation in a deep brain region using Nano gas vesicles as actuators. Adv, Sci. 8:2101934. doi: 10.1002/advs.202101934 PMC8564444 34546652

[ref26] Hu Z. Yang Y. Yang L. Gong Y. Chukwu C. Ye D.. (2024). Airy-beam holographic sonogenetics for advancing neuromodulation precision and flexibility. Proc. Natl. Acad. Sci. 121:e2402200121. doi: 10.1073/pnas.2402200121, PMID: 38885384 PMC11214095

[ref27] Hur S. Choi H. Yoon G. H. Kim N. W. Lee D. G. Kim Y. T. (2022). Planar ultrasonic transducer based on a metasurface piezoelectric ring array for subwavelength acoustic focusing in water [J]. Sci. Rep. 12:1485. doi: 10.1038/s41598-022-05547-7, PMID: 35087151 PMC8795180

[ref28] Ibsen S. Tong A. Schutt C. Esener S. Chalasani S. H. (2015). Sonogenetics is a non-invasive approach to activating neurons in *Caenorhabditis elegans*. Nat. Commun. 6:8264. doi: 10.1038/ncomms9264, PMID: 26372413 PMC4571289

[ref29] Jiang X. He J. J. Zhang C. X. Zhao H. L. Wang W. Q. Ta D. A.. (2022). Three-dimensional ultrasound subwavelength arbitrary focusing with broadband sparse metalens. Sci. China Physics Mechan. Astron. 65:224311. doi: 10.1007/s11433-021-1784-3

[ref30] Jin P. Jan L. Y. Jan Y. N. (2020). Mechanosensitive ion channels: structural features relevant to mechanotransduction mechanisms. Annu. Rev. Neurosci. 43, 207–229. doi: 10.1146/annurev-neuro-070918-050509 32084327

[ref31] Jin Y. Kumar R. Poncelet O. Mondain-Monval O. Brunet T. (2019). Flat acoustics with soft gradient-index metasurfaces. Nat. Commun. 10:143. doi: 10.1038/s41467-018-07990-5, PMID: 30635556 PMC6329837

[ref32] Kim H. Chiu A. Lee S. D. Fischer K. Yoo S. S. (2014). Focused ultrasound-mediated non-invasive brain stimulation: examination of sonication parameters. Brain Stimul. 7, 748–756. doi: 10.1016/j.brs.2014.06.011, PMID: 25088462 PMC4167941

[ref33] Kim Y. G. Kim S. E. Lee J. Hwang S. Yoo S. S. Lee H. W. (2022). Neuromodulation using transcranial focused ultrasound on the bilateral medial prefrontal cortex. J. Clin. Med. 11:3809. doi: 10.3390/jcm11133809 PMC9267901 35807094

[ref34] King R. L. Brown J. R. Newsome W. T. Pauly K. B. (2013). Effective parameters for ultrasound-induced in vivo neurostimulation. Ultrasound Med. Biol. 39, 312–331. doi: 10.1016/j.ultrasmedbio.2012.09.009, PMID: 23219040

[ref35] Kubanek J. Shi J. Marsh J. Chen D. Deng C. Cui J. (2016). Ultrasound modulates ion channel currents. Sci. Rep. 6:24170. doi: 10.1038/srep24170, PMID: 27112990 PMC4845013

[ref36] Kwon D. H. Zhang F. Fedor J. G. Suo Y. Lee S. Y. (2022). Vanilloid-dependent TRPV1 opening trajectory from cryoem ensemble analysis. Nat. Commun. 13:2874. doi: 10.1038/s41467-022-30602-2, PMID: 35610228 PMC9130279

[ref37] Landhuis E. (2017). Ultrasound for the brain [J]. Nature 551, 257–259. doi: 10.1038/d41586-017-05479-7 29120442

[ref38] Lea-Banks H. Meng Y. Wu S. K. Belhadjhamida R. Hamani C. Hynynen K. (2021). Ultrasound-sensitive nanodroplets achieve targeted neuromodulation. J. Control. Release 332, 30–39. doi: 10.1016/j.jconrel.2021.02.010, PMID: 33600879 PMC8089063

[ref39] Lee W. Kim H. Jung Y. Song I. U. Chung Y. A. Yoo S. S. (2015). Image-guided transcranial focused ultrasound stimulates human primary somatosensory cortex. Sci. Rep. 5:5. doi: 10.1038/srep08743 PMC4348665 25735418

[ref40] Lee W. Lee S. D. Park M. Y. Foley L. Purcell-Estabrook E. Kim H.. (2016). Image-guided focused ultrasound-mediated regional brain stimulation in sheep. Ultrasound Med. Biol. 42, 459–470. doi: 10.1016/j.ultrasmedbio.2015.10.001, PMID: 26525652

[ref41] Legon W. Sato T. F. Opitz A. Mueller J. Barbour A. Williams A.. (2014). Transcranial focused ultrasound modulates the activity of primary somatosensory cortex in humans. Nat. Neurosci. 17, 322–329. doi: 10.1038/nn.3620, PMID: 24413698

[ref42] Li J. Liu S. Song C. Hu Q. Zhao Z. Deng T.. (2021). Piezo2 mediates ultrasonic hearing via cochlear outer hair cells in mice. Proc. Natl. Acad. Sci. USA 118:e2101207118. doi: 10.1073/pnas.2101207118 PMC8285978 34244441

[ref43] Li G. F. Zhao H. X. Zhou H. Yan F. Wang J. Y. Xu C. X.. (2016). Improved anatomical specificity of non-invasive neuro-stimulation by high frequency (5 Mhz) ultrasound. Sci. Rep. 6:24738. doi: 10.1038/srep24738, PMID: 27093909 PMC4837374

[ref44] Liao D. Hsiao M. Y. Xiang G. Zhong P. (2021). Optimal pulse length of in sonification for Piezo1 activation and intracellular calcium response. Sci. Rep. 11:709. doi: 10.1038/s41598-020-78553-2, PMID: 33436695 PMC7804118

[ref45] Ma F. Y. Chen J. Y. Wu J. H. Jia H. (2020). Realizing broadband sub-wavelength focusing and a high intensity enhancement with a space-time synergetic modulated acoustic prison. J. Mater. Chem. C 8, 9511–9519. doi: 10.1039/D0TC01984D

[ref46] Martinac B. Buechner M. Delcour A. H. Adler J. Kung C. (1987). Pressure-sensitive ion channel in *Escherichia coli*. Proc. Natl. Acad. Sci. USA 84, 2297–2301. doi: 10.1073/pnas.84.8.2297, PMID: 2436228 PMC304637

[ref47] Marzo A. Caleap M. Drinkwater B. W. (2018). Acoustic virtual vortices with tunable orbital angular momentum for trapping of Mie particles. Phys. Rev. Lett. 120:044301. doi: 10.1103/PhysRevLett.120.044301, PMID: 29437423

[ref48] Marzo A. Seah S. A. Drinkwater B. W. Sahoo D. R. Long B. Subramanian S. (2015). Holographic acoustic elements for manipulation of levitated objects. Nat. Commun. 6:8661. doi: 10.1038/ncomms9661, PMID: 26505138 PMC4627579

[ref49] Meng L. Liu X. Wang Y. Zhang W. Zhou W. Cai F.. (2019). Sonoporation of cells by a parallel stable cavitation microbubble Array. Advanced Sci. 6:1900557. doi: 10.1002/advs.201900557 PMC6724477 31508275

[ref50] Mueller J. Legon W. Opitz A. Sato T. F. Tyler W. J. (2014). Transcranial focused ultrasound modulates intrinsic and evoked EEG dynamics. Brain Stimul. 7, 900–908. doi: 10.1016/j.brs.2014.08.008, PMID: 25265863

[ref51] O'brien W. D. (2007). Ultrasound-biophysics mechanisms. Prog. Biophys. Mol. Biol. 93, 212–255. doi: 10.1016/j.pbiomolbio.2006.07.010, PMID: 16934858 PMC1995002

[ref52] Oh S. J. Lee J. M. Kim H. B. Lee J. Han S. Bae J. Y.. (2019). Ultrasonic neuromodulation via astrocytic Trpa1. Curr. Biol. 29, 3386–401.e8. doi: 10.1016/j.cub.2019.08.021, PMID: 31588000

[ref53] Pan Y. Yoon S. Sun J. Huang Z. Lee C. Allen M.. (2018). Mechanogenetics for the remote and noninvasive control of cancer immunotherapy. Proc. Natl. Acad. Sci. USA 115, 992–997. doi: 10.1073/pnas.1714900115, PMID: 29343642 PMC5798350

[ref54] Pan X. Zeng L. Li Y. Zhu X. Jin Y. (2023). Experimental demonstration of Fresnel zone plate lens for robust subwavelength focusing at mega hertz. Ultrasonics 128:106876. doi: 10.1016/j.ultras.2022.106876, PMID: 36272298

[ref55] Park M. Hoang G. M. Nguyen T. Lee E. Jung H. J. Choe Y.. (2021). Effects of transcranial ultrasound stimulation pulsed at 40 Hz on Aβ plaques and brain rhythms in 5xFAD mice. Transl. Neurodegen. 10:48. doi: 10.1186/s40035-021-00274-x PMC8650290 34872618

[ref56] Pasquinelli C. Hanson L. G. Siebner H. R. Lee H. J. Thielscher A. (2019). Safety of transferential focused ultrasound stimulation: a systematic review of the state of knowledge from both human and animal studies. Brain Stimul. 12, 1367–1380. doi: 10.1016/j.brs.2019.07.024, PMID: 31401074

[ref57] Qi S. Li Y. Assouar B. (2017). Acoustic focusing and energy confinement based on multilateral metasurfaces. Physical Rev. Appl. 7:054006. doi: 10.1103/PhysRevApplied.7.054006

[ref58] Qiu Z. Guo J. Kala S. Zhu J. Xian Q. Qiu W.. (2019). The mechanosensitive Ion Channel Piezo1 significantly mediates in vitro ultrasonic stimulation of neurons. iScience 21, 448–457. doi: 10.1016/j.isci.2019.10.037, PMID: 31707258 PMC6849147

[ref59] Qiu Z. Kala S. Guo J. Xian Q. Zhu J. Zhu T.. (2021). Targeted neurostimulation in mouse brains with non-invasive ultrasound. Cell Rep. 34:108595. doi: 10.1016/j.celrep.2020.108595, PMID: 33406437

[ref60] Romero M. C. Davare M. Armendariz M. Janssen P. (2019). Neural effects of transcranial magnetic stimulation at the single-cell level. Nat. Commun. 10:2642. doi: 10.1038/s41467-019-10638-7, PMID: 31201331 PMC6572776

[ref61] Salling M. C. Martinez D. (2016). Brain stimulation in addiction. Neuropsychopharmacology 41, 2798–2809. doi: 10.1038/npp.2016.80, PMID: 27240657 PMC5061891

[ref62] Sanchis L. Yánez A. Galindo P. L. Pizarro J. Pastor J. M. (2010). Three-dimensional acoustic lenses with axial symmetry. Appl. Phys. Lett. 97:054103. doi: 10.1063/1.3474616

[ref63] Sato T. Shapiro M. G. Tsao D. Y. (2018). Ultrasonic neuromodulation causes widespread cortical activation via an indirect auditory mechanism. Neuron 98, 1031–41.e5. doi: 10.1016/j.neuron.2018.05.00 29804920 PMC8127805

[ref64] Servick K. (2020). Hope grows for targeting the brain with ultrasound. Science 368, 1408–1409. doi: 10.1126/science.368.6498.1408, PMID: 32586996

[ref65] Sharabi S. Daniels D. Last D. Guez D. Zivli Z. Castel D.. (2019). Non-thermal focused ultrasound induced reversible reduction of essential tremor in a rat model. Brain Stimul. 12, 1–8. doi: 10.1016/j.brs.2018.08.014, PMID: 30181107

[ref66] Shen X. Song Z. Xu E. Zhou J. Yan F. (2021). Sensitization of nerve cells to ultrasound stimulation through Piezo1-targeted microbubbles [J]. Ultrason. Sonochem. 73:105494. doi: 10.1016/j.ultsonch.2021.105494, PMID: 33640571 PMC7921623

[ref67] Sorum B. Rietmeijer R. A. Gopakumar K. Adesnik H. Brohawn S. G. (2021). Ultrasound activates mechanosensitive TRAAK K(+) channels through the lipid membrane. Proc. Natl. Acad. Sci. USA 118:e2006980118. doi: 10.1073/pnas.2006980118 PMC8017979 33542098

[ref68] Tarrazó-Serrano D. Pérez-López S. Candelas P. Uris A. Rubio C. (2019). Acoustic focusing enhancement in Fresnel zone plate lenses [J]. Sci. Rep. 9:7067. doi: 10.1038/s41598-019-43495-x, PMID: 31068613 PMC6506507

[ref69] Tarrazó-Serrano D. Rubio C. Minin O. V. Candelas P. Minin I. V. (2019). Manipulation of focal patterns in acoustic Soret type zone plate lens by using reference radius/phase effect. Ultrasonics 91, 237–241. doi: 10.1016/j.ultras.2018.07.022, PMID: 30126723

[ref70] Tufail Y. Matyushov A. Baldwin N. Tauchmann M. L. Georges J. Yoshihiro A.. (2010). Transcranial pulsed ultrasound stimulates intact brain circuits. Neuron 66, 681–694. doi: 10.1016/j.neuron.2010.05.008, PMID: 20547127

[ref71] Tyler W. J. (2011). Noninvasive neuromodulation with ultrasound? A continuum mechanics hypothesis. Neuroscientist 17, 25–36. doi: 10.1177/1073858409348066, PMID: 20103504

[ref72] Tyler W. J. Lani S. W. Hwang G. M. (2018). Ultrasonic modulation of neural circuit activity. Curr. Opin. Neurobiol. 50, 222–231. doi: 10.1016/j.conb.2018.04.011 29674264

[ref73] Wagner T. Valero-Cabre A. Pascual-Leone A. (2007). Noninvasive human brain stimulation [J]. Annu. Rev. Biomed. Eng. 9, 527–565. doi: 10.1146/annurev.bioeng.9.061206.133100 17444810

[ref74] Wang X. Yan J. Wang Z. Li X. Yuan Y. (2020). Neuromodulation effects of ultrasound stimulation under different parameters on mouse motor cortex. I.E.E.E. Trans. Biomed. Eng. 67, 291–297. doi: 10.1109/TBME.2019.2912840 31021758

[ref75] Wattiez N. Constans C. Deffieux T. Daye P. M. Tanter M. Aubry J. F.. (2017). Transcranial ultrasonic stimulation modulates single-neuron discharge in macaques performing an antisaccade task. Brain Stimul. 10, 1024–1031. doi: 10.1016/j.brs.2017.07.007, PMID: 28789857

[ref76] Wu J. Goyal R. Grandl J. (2016). Localized force application reveals mechanically sensitive domains of Piezo1. Nat. Commun. 7:12939. 27694883 10.1038/ncomms12939 PMC5063965

[ref77] Wu X. Xia X. Tian J. Liu Z. Wen W. (2016). Broadband reflective metasurface for focusing underwater ultrasonic waves with linearly tunable focal length. Appl. Phys. Lett. 108:163502. doi: 10.1063/1.4947437

[ref78] Xia X. X. Li Y. C. Cai F. Y. Zhou H. Ma T. Zheng H. R. (2020). Ultrasonic tunable focusing by a stretchable phase-reversal Fresnel zone plate. Appl. Phys. Lett. 117:021904. doi: 10.1063/5.0018663

[ref79] Yang Y. Yuan J. Field R. L. Ye D. Hu Z. Xu K.. (2023). Induction of a torpor-like hypothermic and hypometabolic state in rodents by ultrasound. Nat. Metab. 5, 789–803. doi: 10.1038/s42255-023-00804-z, PMID: 37231250 PMC10229429

[ref80] Ye P. P. Brown J. R. Pauly K. B. (2016). Frequency dependence of ultrasound Neurostimulation in the mouse brain. Ultrasound Med. Biol. 42, 1512–1530. doi: 10.1016/j.ultrasmedbio.2016.02.012, PMID: 27090861 PMC4899295

[ref81] Ye J. Tang S. Meng L. Li X. Wen X. Chen S.. (2018). Ultrasonic control of neural activity through activation of the Mechanosensitive Channel MscL. Nano Lett. 18, 4148–4155. doi: 10.1021/acs.nanolett.8b00935, PMID: 29916253

[ref82] Yildiz D. Göstl R. Herrmann A. (2022). Sonopharmacology: controlling pharmacotherapy and diagnosis by ultrasound-induced polymer mechanochemistry. Chem. Sci. 13, 13708–13719. doi: 10.1039/d2sc05196f 36544723 PMC9709924

[ref83] Yoo S. S. Bystritsky A. Lee J. H. Zhang Y. Fischer K. Min B. K.. (2011). Focused ultrasound modulates region-specific brain activity [J]. NeuroImage 56, 1267–1275. doi: 10.1016/j.neuroimage.2011.02.058, PMID: 21354315 PMC3342684

[ref84] Yoo S. Mittelstein D. R. Hurt R. C. Lacroix J. Shapiro M. G. (2022). Focused ultrasound excites cortical neurons via mechanosensitive calcium accumulation and ion channel amplification. Nat. Commun. 13:493. doi: 10.1038/s41467-022-28040-1, PMID: 35078979 PMC8789820

[ref85] Yoon K. Lee W. Lee J. E. Xu L. Croce P. Foley L.. (2019). Effects of sonication parameters on transcranial focused ultrasound brain stimulation in an ovine model. PLoS One 14:e0224311. doi: 10.1371/journal.pone.0224311, PMID: 31648261 PMC6812789

[ref86] Yu K. Sohrabpour A. He B. (2016). Electrophysiological source imaging of brain networks perturbed by low-intensity transcranial focused ultrasound. I.E.E.E. Trans. Biomed. Eng. 63, 1787–1794. doi: 10.1109/TBME.2016.2591924 PMC5247426 27448335

[ref87] Zhang L. Liu X. Gao L. Ji Y. Wang L. Zhang C.. (2020). Activation of Piezo1 by ultrasonic stimulation and its effect on the permeability of human umbilical vein endothelial cells. Biomed. Pharmacother. 131:110796. doi: 10.1016/j.biopha.2020.110796, PMID: 33152952

[ref88] Zhou X. Assouar M. B. Oudich M. (2014). Acoustic superfocusing by solid phononic crystals. Appl. Phys. Lett. 105:233506. doi: 10.1063/1.4904262

[ref89] Zhu J. Xian Q. Hou X. Wong K. F. Zhu T. Chen Z.. (2023). The mechanosensitive ion channel Piezo1 contributes to ultrasound neuromodulation. Proc. Natl. Acad. Sci. USA 120:e2300291120. doi: 10.1073/pnas.2300291120, PMID: 37098060 PMC10161134

